# Whole genome sequencing of Nontuberculous Mycobacterium (NTM) isolates from sputum specimens of co-habiting patients with NTM pulmonary disease and NTM isolates from their environment

**DOI:** 10.1186/s12864-020-6738-2

**Published:** 2020-04-23

**Authors:** Jung-Ki Yoon, Taek Soo Kim, Jong-Il Kim, Jae-Joon Yim

**Affiliations:** 1 Division of Pulmonary and Critical Care Medicine, Department of Internal Medicine, Seoul National University College of Medicine, Seoul, Republic of Korea; 2 Department of Laboratory Medicine, Seoul National University Hospital, Seoul, Republic of Korea; 3 Department of Biomedical Sciences, Seoul National University Graduate School, Seoul, Republic of Korea; 4 Genomic Medicine Institute, Medical Research Center, Seoul National University School, Seoul, Republic of Korea

**Keywords:** Non-tuberculosis mycobacterium, Whole genome sequencing, Transmission, Non-tuberculous mycobacterial pulmonary disease, Phylogenomics

## Abstract

**Background:**

Nontuberculous mycobacterium (NTM) species are ubiquitous microorganisms. NTM pulmonary disease (NTM-PD) is thought to be caused not by human-to-human transmission but by independent environmental acquisition. However, recent studies using next-generation sequencing (NGS) have reported trans-continental spread of *Mycobacterium abscessus* among patients with cystic fibrosis.

**Results:**

We investigated NTM genomes through NGS to examine transmission patterns in three pairs of co-habiting patients with NTM-PD who were suspected of patient-to-patient transmission. Three pairs of patients with NTM-PD co-habiting for at least 15 years were enrolled: a mother and a daughter with *M. avium-*PD, a couple with *M. intracellulare-*PD, and a second couple, one of whom was infected with *M. intracellulare* and the other of whom was infected with *M. abscessus*. Whole genome sequencing was performed using patients’ NTM isolates as well as environmental specimens. Genetic distances were estimated based on single nucleotide polymorphisms (SNPs). By comparison with the genetic distances among 78 publicly available NTM genomes, NTM isolates derived from the two pairs of patients infected with the same NTM species were not closely related to each other. In phylogenetic analysis, the NTM isolates from patients with *M. avium*-PD clustered with isolates from different environmental sources.

**Conclusions:**

In conclusion, considering the genetic distances between NTM strains, the likelihood of patient-to-patient transmission in pairs of co-habiting NTM-PD patients without overt immune deficiency is minimal.

## Background

The prevalence of nontuberculous mycobacterial pulmonary disease (NTM-PD) is increasing in developed countries [1–4]. Several explanations for this epidemiological change have been proposed, including awareness and improved detection of NTM-PD, increased populations with risk factors such as bronchiectasis or use of immunosuppressants, and disinfection of drinking water in urban areas resulting in selective advantages for NTM [1].

The development of next-generation sequencing (NGS) technology enables the identification of massive numbers of single nucleotide polymorphisms (SNPs) by whole genome sequencing (WGS). Using SNPs as genetic fingerprints and comparing them among multiple samples, phylogenetic analysis has been able to identify the source of infection for pathogens such as *Vibrio cholera* [5], *Staphylococcus aureus* [6], *Pseudomonas aeruginosa* [7], and NTM [8–11].

Since NTM are ubiquitous microorganism, it is generally assumed that patients with NTM-PD acquire NTM from their environment, not from other infected individuals. However, recent NGS studies showed that this might not be the case, at least for patients with cystic fibrosis [8, 9]. Bryant and colleagues collected *Mycobacterium abscessus* isolates from patients with cystic fibrosis, performed WGS, and analysed phylogenetic relationships among these isolates. Surprisingly, they found strong evidence supporting human-to-human transmission of *M. abscessus* among patients with cystic fibrosis, and identified some *M. abscessus* isolates that were widespread globally [9]. Although Harris and colleagues reported no evidence of patient-to-patient transmission in their cohort of pediatric cystic fibrosis patients [10], NGS studies of NTM transmission have raised concerns that NTM might be transmitted not only among immunosuppressed individuals but also among immunocompetent ones. This would be especially important for hospital infection control, since isolation practices for NTM-PD patients without cystic fibrosis are not as strict as those for pulmonary tuberculosis patients generally.

Recently, we diagnosed three pairs of NTM-PD patients with no immunodeficiency who had been co-habiting for at least 15 years. We investigated the genomes of NTM isolates derived from the patients and environmental samples in their houses to understand the source of infection using WGS.

## Results

### Patient characteristics

Three pairs of patients with NTM-PD who had been co-habiting for at least 15 years were enrolled (Table 1). A mother and a daughter (Patients A and B) with *M. avium* PD had lived in an apartment in an urban area (HOME-1) for 15 years. A couple (Patients C and D) with *M. intracellulare* PD had lived in a house in a rural area (HOME-2) for 30 years. A second couple (Patients E and F) with *M. intracellulare* PD and *M. abscessus* subsp. *massiliense* PD had lived in an apartment in an urban area (HOME-3) for 30 years. No patients were suspected of any immunodeficiency disorders. They were HIV-negative, were not taking any immunosuppressants and had no history of recurrent infection of any organs.

**Table 1:** Characteristics of the three patient pairs in this study

	Age /Relationship	Sources	NTM Collection Date	NTM Species by conventional method	Habitat	Radiologic findings
Patient A	81 /Mother	Sputum	*5 June 2017*	*M. avium*	HOME-1 Apartment (urban area) for 15 years.	Bronchiectasis and centrilobular nodules in RML/LUL lingular segments
Patient B	51 /Daughter	Sputum	*19 July 2017*	*M. avium*		Centrilobular nodules with branching opacity in RUL/RML/LUL lingula segments
Patient C	77 /Husband	Sputum	*13 April 2017*	*M. intracellulare*	HOME-2 House (rural area) for 30 years with high soil environment	Lung nodule in RUL
Patient D	71 /Wife	Sputum	*3 May 2017*	*M. intracellulare*		Multiple branching opacity and centrilobular nodules in both lung
Patient E	62 /Husband	Bronchial washing	*29 September 2017*	*M. intracellulare*	HOME-3 Apartment (urban area) for 30 years.	Bronchiectasis with peribonchial infiltration in LLL
Patient F	61 /Wife	Bronchial washing	*29 September 2017*	*M. abscessus* subsp. *massiliense*		Patchy opacity and nodules in RML

*RUL* right upper lobe, *RML* right middle lobe, *LUL* left upper lobe, *LLL* left lower lobe

### NTM isolation and sequencing

Among 12 environmental specimens from HOME-1, seven specimens from either the kitchen or the bathroom were culture-positive. Subsequently, 18 morphologically distinct isolates were purified (Supplementary Table 1, Additional File 1). However, none of the 15 environmental specimens from HOME-2 yielded any NTM isolates and only one of 15 specimens from HOME-3 yielded NTM isolates.

On average, 19.8 million sequencing reads were obtained for each isolate (See Supplementary Table 2, Additional File 1). According to *k*-mer based taxonomic classification of NGS reads, 12 isolates from environmental specimens in HOME-1, isolates from Patient A and Patient B were identified as *M. avium* subsp. *hominissuis*. Isolates from Patients C, D, and E were identified as *M. intracellulare*, and one isolate from Patient F was identified as *M. abscessus*. The identifications of patient-derived isolates using NGS produced the same results as conventional PCR and direct sequencing. Two isolates from environmental specimens in HOME-1 were identified as *M. fortuitum*, while the remaining four isolates from HOME-1 and three isolates from HOME-3 were classified as non-NTM and were excluded.

The mean sequencing depth of identified isolates was 313× (210–487×). To reduce the potential impact of recombination and mobile genetic elements on our results, we defined core regions for each species as common sequences observed across the 17 *M. avium* subsp. *hominissuis* and 3 *M. intracellulare* genomes analyzed in this study (as well as 64 and 14 publicly available genomes, respectively). The core region of *M. avium* subsp. *homonissuis* consisted of 4.30 Mbp of the 5.15 Mbp genome (83%), and that of *M. intracellulare* consisted of 4.23 Mbp of the 5.40 Mbp genome (78%). On average, 38,377 (27,469–43,720) high confidence SNPs were detected in *M. avium* subsp. *hominissuis* and 16,464 (15–26,740) high confidence SNPs were detected in *M. intracellulare* within these regions (Supplementary Table 2, Additional File 1). Based on the distributions of SNP allele fraction (Supplementary Table 2, Additional File 1), all isolates were monoclonal except for the isolate from Patient C, which consisted of two clones at an 8:2 ratio (Supplementary Figure 1). The 25,441 and 2485 high confidence SNPs from Patient C were classified as belonging to the Cmajor and Cminor clones based on SNP allele fractions.

### Pairwise SNP distances and phylogenetic analysis

**Fig. 1:**
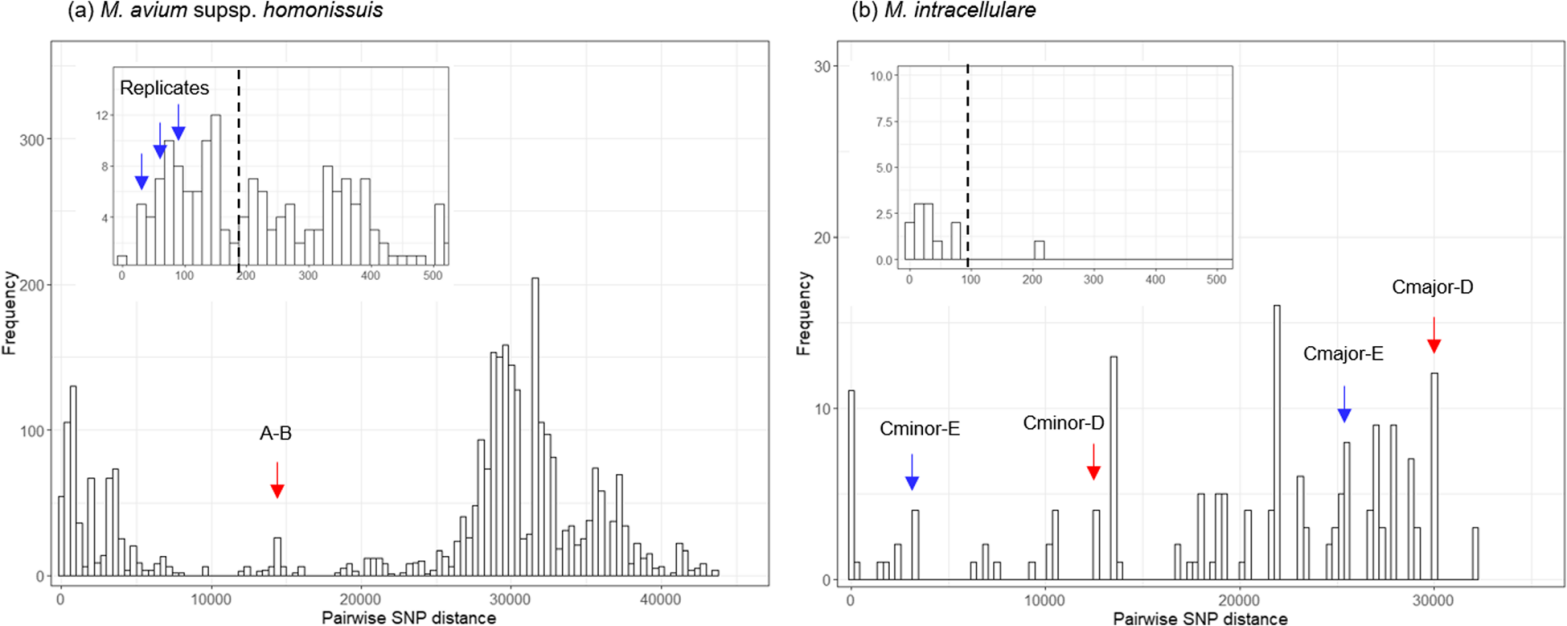
Histograms of pairwise SNP distances. The x-axis shows pairwise SNP distance and the y-axis shows the frequencies of distances between isolates. (**a**) Distances between replicates of *M. avium* subsp. *hominissuis* (blue arrow) were less than 200 (black dashed line), whereas the distance between isolates from Patient A and B (red arrow) was 14,768. (**b**) The distance between *M. intracellulare* isolates from Patients C and D living together in HOME-2 (red arrow) was higher than the distance between isolates from Patients C and E living in other houses (blue arrow)

**Fig. 2:**
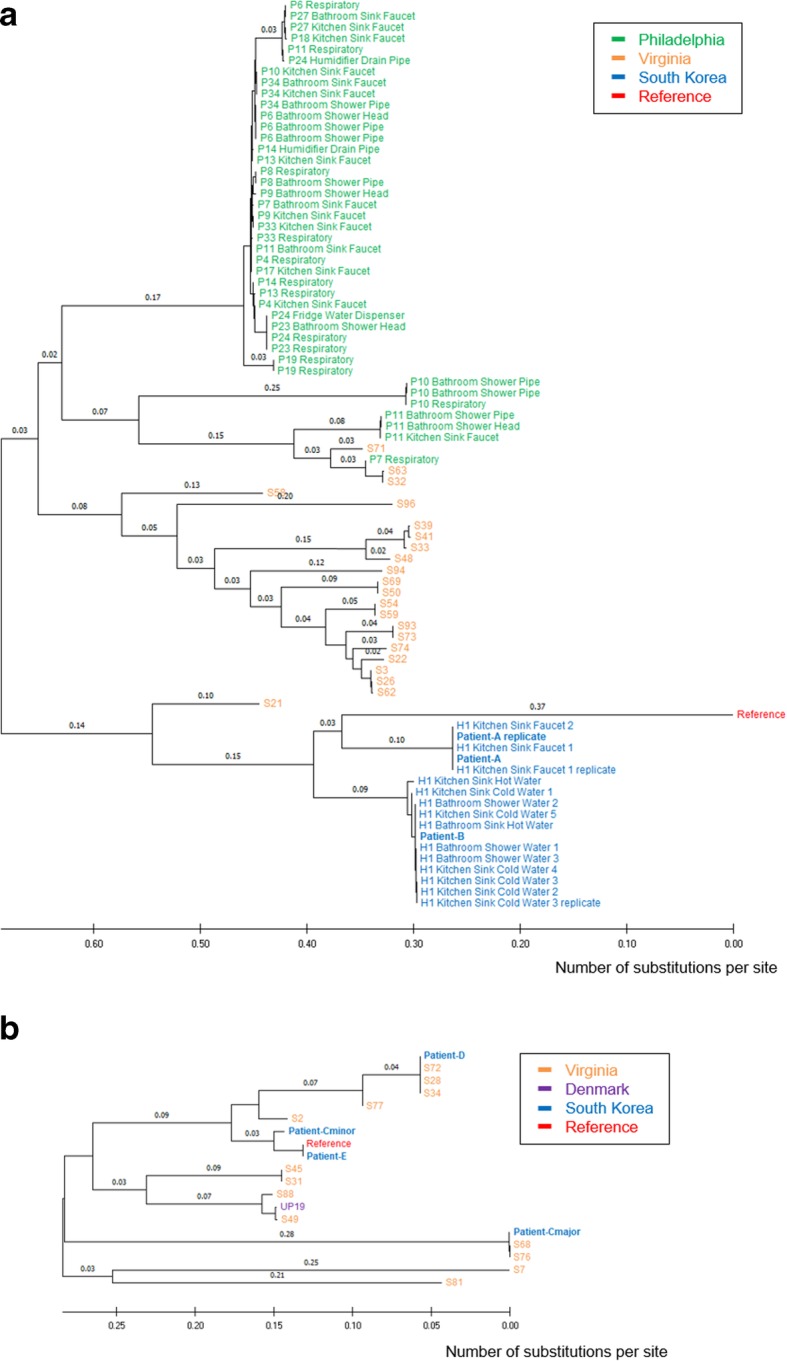
Phylogenetic analysis of *M. avium* subsp. *homonissuis* and *M. intracellulare* isolates. A neighbor-joining phylogenetic tree was drawn to scale with branch lengths in units of number of base substitutions per site. (**a**) Isolates from Patients A and B (blue) clustered apart from environmental isolates from HOME-1, and 64 publicly available *M. avium* subsp. *hominissuis* genome sequences clustered as expected based on previously published work. (**b**) Isolates from Patients C and D (blue) clustered separately from other isolates

A total of 104,531 and 102,281 genomic positions with high-confidence SNPs were identified in *M. avium* subsp. *hominissuis* and *M intracellulare* genome, respectively, and used for further analysis. Pairwise SNP distances between every pair of isolates at those positions were calculated and clusters on the histogram of SNP distances were observed (Fig. 1). The SNP distance between the isolates from Patient A and B was 14,768. By contrast, the SNP distances among three replicates (the isolates from Patient A, Kitchen Sink Faucet 1, and Kitchen Sink Cold Water 3) were less than 100. (Fig. 1a) Using high-confidence SNPs from 81 *M. avium* subsp. *hominissuis* genomes, phylogenetic analysis was performed (Fig. 2a). The isolate from Patient A and its replicates clustered with the specimens from the kitchen (scale on surface of kitchen faucet), while the isolate from Patient B clustered with the isolates from the bathroom (hot water from bathroom faucet, hot water from showerhead) and the kitchen (cold water from kitchen faucet).

In HOME-2, the SNP distance between the *M. intracellulare* isolates from Patients Cmajor and D was 29,873, and the distance between Patient Cminor and D was 12,670. Those distances were even higher than the SNP distances from Patient E in HOME-3. (Fig. 1b) Phylogenetic analysis with 17 *M. intracellulare* genomes confirmed that all three isolates from Patient C, D, and E were not closely related each other. (Fig. 2b) In HOME-3, different species of NTM were isolated (*M. intracellulare* from Patient E and *M. abscessus* subsp. *massiliense* from patient F), and the SNP distance was not calculated.

## Discussion

Recent NGS studies suggest the patient-to-patient transmission of *M. abscessus* among cystic fibrosis patients [8, 9]. In response to these findings, the NTM guidelines have been updated. The British Thoracic Society [12], US Cystic Fibrosis Foundation and European Cystic Fibrosis Society [13] recommended that infection control policies minimize risks of patient-to-patient transmission for patients with both cystic fibrosis and *M. abscessus*-PD. However, these guidelines were only designed for patients with underlying risk factors and contain no statements for patients without immunodeficiencies. In this context, it is clinically important to understand if there is any evidence of patient-to-patient transmission among patients without predisposing factors.

Among the three pairs of co-habiting NTM-PD patients without overt immunodeficiency examined here, no patient pairs had near-identical NTM isolates (Fig. 2). A pair of patients were infected with *M. avium*-PD isolates that were genetically distinct but similar to separate isolates from environmental specimens in their homes. Although we could not identify environmental sources for the remaining two pairs of patients, one patient pair was also infected with genetically distinct *M. intracellulare* isolates (SNP distance > 10^4^), and the last pair of patients was infected by different species of NTM (*M. intracellulare* and *M. abscessus* subsp. *massiliense*). Therefore, patient-to-patient transmission is less likely in our study.

Household water sources are considered a major reservoir for NTM, especially for *M. avium*. One report showed that samples from 17 (46%) of 37 households yielded the same species of NTM found in the patient. In seven of those households, the patient isolate and a plumbing isolate exhibited the same repetitive sequence-based PCR DNA fingerprint. Another study reported that seven of 20 (35%) patients with NTM-PD had NTM isolates with identical genotypes to isolates from household water or shower aerosols [14]. Recently, variable number tandem repeat genotyping and WGS revealed that 11 of 21 (52%) patients with *M. avium*-PD had genetically matched isolates to household isolates [11]. In our study, a phylogenetic tree based on WGS was consistent with these previous reports and demonstrated the environmental sources of two *M. avium*-PD patients living together (HOME-1). Patient A seemed to have acquired NTM from the kitchen, while Patient B acquired NTM from the bathroom or kitchen.

Although we thoroughly collected environmental specimens from water and biofilms on the faucets and showerhead, we could not culture any NTMs from HOME-2, and identified only one NTM from HOME-3. A previous study reported that NTM species could be isolated from 22 of 37 (59%) households of NTM-PD patients. In addition, a positive correlation was observed between the number of samples collected per house and the number of NTM-positive samples [15]. We collected 15 water samples from each of the two houses; this number is equal to or larger than that of most studies, suggesting that there may be sources of NTM-PD other than water. The patients living in HOME-2 (Patient C and D) were farmers and their home was a high soil environment that included a yard and hay (Table 1). Given that previous studies reported that a majority of *M. intracellulare* was isolated from soil samples, especially in high soil environments [14, 16, 17], the source of *M. intracellulare* for these patients could be soil instead of water.

In HOME-3, three morphologically distinct colonies were cultured from one environmental specimen and sequenced separately. However, using *k*-mer based taxonomic classification, these isolates were not NTM. The sequencing reads of NTM isolates from Patients E and F aligned to NTM reference genomes, but those from environmental specimen isolates did not. Thus, these environmental isolates were not closely related to NTM in HOME-3. A previous study showed that, among 19 patients with *M. avium*-PD or *M. intracellulare*-PD living in low soil environments, no patients had genetically identical isolates compared with soil sample isolates from their houses [17]. Our results suggested that a patient with *M. intracellulare*-PD (Patient E) in HOME-3 (low soil) may have been exposed to the NTM outside the house. The lag time between the acquisition of NTM from the environment and diagnosis of NTM-PD also makes it more difficult to identify the source of NTM [14].

## Conclusions

NTM isolates derived from three pairs of co-habiting NTM-PD patients were determined to be different strains based on WGS data. The sources of *M. avium* in patients in one pair was identified: one acquired infection from the kitchen and the other from the bathroom or kitchen. The likelihood of patient-to-patient transmission appeared minimal in these three pairs of NTM-PD patients.

## Methods

### Patient enrolment

Pairs of adult patients living together and diagnosed with NTM-PD at Seoul National University Hospital satisfying the following inclusion criteria were enrolled: age > 18 years; typical respiratory symptoms such as chronic cough, sputum, or haemoptysis; findings suggestive of NTM-PD on computed tomography; identification of NTM in ≥2 sputum cultures or in ≥1 bronchoscopic specimen; living in the same household prior to diagnosis with NTM-PD; and consented to collection of environmental samples in their home. This study was approved by an institutional review board (IRB Number: 1804–064-936) and registered at ClinicalTrial.gov (NCT03532438).

### Sample collection and NTM isolation

The most recently-isolated NTM from participant sputum or bronchial wash specimens from participants was collected. In addition, we visited patients’ homes to collect environmental specimens. One litre of water was aseptically collected into sterile containers directly from all faucets and showers in bathrooms and kitchens. Swabs were taken from the inside of faucets and showerheads in bathrooms and kitchens. As previously described [14, 18], each water specimen was passed through a 0.45 μm filter. Filters were rinsed with 10 mL of sterile distilled water and transferred to Middlebrook 7H11 plates and Mycobacterial Growth Indicator Tubes (MGITs). Swabs were washed in sterile distilled water and then processed in the same manner as the water samples. All culture-positive MGITs were transferred to new Middlebrook 7H11 plates. If culture-positive Middlebrook 7H11 plates had two or more morphologically distinct colonies in terms of size and color, each distinct colony was separately transferred to a 3% Ogawa media plate, incubated, and purified as a single colony. If the colonies were homogenous and discrete on Middlebrook 7H11 plates, we collected the isolates without a further purification step.

### DNA preparation and sequencing

All biomass from Middlebrook 7H11 plates or Ogawa media plates taken from sweeps of colonies was mixed with 425–600 μm glass beads, vortexed for 5 min, incubated at 80 °C for 10 min and then centrifuged. DNA was extracted from the supernatant using a QIAamp DNA mini kit (Qiagen inc, Hilden, Germany) as previously described [8]. Subsequently, DNA was sequenced using an Illumina HiSeq or NovaSeq instrument (Illumina, San Diego, USA). To validate the method, two patient isolates and an environmental specimen isolate were sequenced twice as replicates. Identification of each isolate from the patients was performed by 16S rRNA [19] and *rpoB* gene [20, 21] sequencing analysis.

### Variant calling and phylogenetic analysis

Kraken2, a taxonomic classification system using *k*-mer matches [22], was used to identify reads from each isolate mapping to reference genomes. For isolates assigned as *M. avium* subsp. *homonissius*, reads were mapped to *M. avium* subsp. *homonissius* TH15 using BWA [23]. For isolates assigned as *M. intracellulare*, reads were mapped to *M. intracellulare* ATCC 13950. Isolates assigned by Kraken2 as non-NTM species were excluded from further analyses. Variants were called using SAMtools and bcftools [24] with the following filters: minimum base quality of 50, minimum mapping quality of 30, support from at least four reads (two forward reads, two reverse reads), and absence of heterozygosity. For comparison, 64 *M. avium* subsp. *homonissius* and 14 *M. intracellulare* genomes available in three publicly available datasets deposited in the short read archive (PRJNA339271, PRJNA506132, PRJEB13214) were downloaded and processed using the same procedures [11, 25, 26]. Core regions were defined for each NTM species as all genomic positions with depths from all isolates of each species of mean depth ± two standard deviations. High-confidence SNPs were defined as SNPs in the core regions of each NTM species. SNP distance and the number of different high-confidence SNPs were calculated for each pair of isolates. The phylogenetic analysis was conducted with MEGA-X (version 10.0.5) [27] using only high confidence SNPs. The genetic distances between isolates were computed using the maximum composite likelihood method and a phylogenetic tree was constructed using neighbor-joining methods. The rate variation among sites was modeled with a gamma distribution (shape parameter = 1). Reliability of the phylogeny was assessed using the bootstrap test (number of bootstrap replicates = 100). All sequence data was deposited in the National Center for Biotechnology Information database as BioProject ID PRJNA577108.

## Supplementary information

**Additional file 1: Table S1.** Characteristics on environmental specimens. The numbers of collected specimens, culture-positive, morphologically distinct isolates from each habitat. **Table S2.** NTM species Identified and Sequencing Results. Description on the source of each sample and its sequencing quality (total read counts, mean depth, 20x coverage, number of SNPs), species, clonality, and morphologic feature.

**Additional file 2.**

## Data Availability

All sequence data supporting the conclusions of this article is available in the National Center for Biotechnology Information database as BioProject ID PRJNA577108.

## Abbreviations

MGIT: Mycobacterial growth indicator tubes
NGS: Next-generation sequencing
NTM: Non-tuberculous mycobacterium
NTM-PD: Non-tuberculous mycobacterial pulmonary disease
SNP: Single nucleotide polymorphism
WGS: Whole genome sequencing
