# Supplementary figures and images for: Whole genome sequencing of Nontuberculous Mycobacterium (NTM) isolates from sputum specimens of co-habiting patients with NTM pulmonary disease and NTM isolates from their environment
Source: BMC Genomics. 2020 Apr 23;21:322. doi: 10.1186/s12864-020-6738-2 (PMC7181514; doi:10.1186/s12864-020-6738-2)

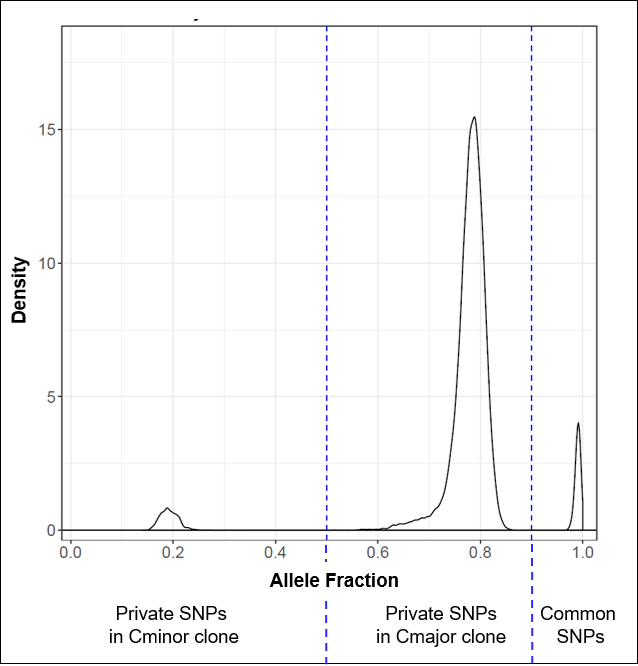

Supplement: Supplementary file 2 — Additional file 2. [file 12864_2020_6738_MOESM2_ESM.tif]
